# Clinical Utility of Ammonia Concentration as a Diagnostic Test in Monitoring of the Treatment with L-Asparaginase in Children with Acute Lymphoblastic Leukemia

**DOI:** 10.1155/2014/945860

**Published:** 2014-07-23

**Authors:** Małgorzata Czogała, Walentyna Balwierz, Krystyna Sztefko, Iwona Rogatko

**Affiliations:** ^1^Department of Pediatric Oncology and Hematology, Children's University Hospital, ul. Wielicka 265, 30-663 Krakow, Poland; ^2^Department of Pediatric Oncology and Hematology, Polish American Institute of Pediatrics, Jagiellonian University Medical College, ul. Wielicka 265, 30-663 Krakow, Poland; ^3^Department of Clinical Biochemistry, Polish American Institute of Pediatrics, Jagiellonian University Medical College, ul. Wielicka 265, 30-663 Krakow, Poland

## Abstract

L-asparaginase (ASP) is an enzyme used as one of the basic regimens in the acute lymphoblastic leukemia (ALL) therapy. Because of the possibility of the enzyme inactivation by antibodies, monitoring of ASP activity is essential. The aim of the study was to examine if plasma concentration of ammonia, a direct product of the reaction catalyzed by ASP, can be used in the assessment of ASP activity. A group of 87 patients with acute lymphoblastic leukemia treated in the Department of Pediatric Oncology and Hematology in Krakow was enrolled to the study. ASP activity and ammonia concentration were measured after ASP administrations during induction. A positive correlation was found between the ammonia concentration and ASP activity (*R* = 0.44; *P* < 0.0001) and between the medium values of ammonia concentration and ASP activity (*R* = 0.56; *P* < 0.0001). The analysis of ROC curves revealed the moderate accuracy of the ammonia concentration values in the ASP activity assessment. It was also found that the medium value of ammonia concentrations can be useful in identification of the patients with low (<100 IU/L) and undetectable (<30 IU/L) ASP activity. The plasma ammonia concentration may reflect ASP activity and can be useful when a direct measurement of the activity is unavailable.

## 1. Introduction

L-asparaginase (ASP), an enzyme catalyzing hydrolysis of asparagine to aspartic acid and ammonia, is one of the basic regimens used in the treatment of acute lymphoblastic leukemia (ALL). Neoplastic blasts have reduced expression of asparagine synthetase and thus need asparagine from the circulating blood [1]. ASP causes asparagine depletion from plasma, leading to inhibition of protein biosynthesis in blast cells, cell cycle arrest, and finally cellular death [2]. The treatment efficacy is related to the duration and grade of the reduction of the asparagine concentration in plasma and cerebrospinal fluid, which depends on the ASP activity. It is considered that the therapeutic ASP activity is above 100 IU/L [3–6] but a complete asparagine depletion was observed in some patients with lower enzyme activity [3, 5, 6].

ASP can be derived from* Escherichia coli* (*E. coli*) or* Erwinia chryzantemi *(*Erwinia*) [7]. There are native and pegylated (PEG-ASP) preparations available, which differ in pharmacokinetics. Distinct treatment schedules are used for each preparation to assure the optimal efficacy in most of the patients.

ASP, as a nonhuman protein, can cause antibodies development. Hypersensitivity can be clinically visible or “silent,” when the drug activity decreases without clinical symptoms [8–12]. The reported frequency of antiasparaginase antibodies is variable and reaches up to 70% [7, 12]. The frequency of hypersensitivity reactions ranges from 0% to 45% [7, 12]. Both situations are an indication for switching to another ASP preparation (pegylated or from another bacterial source) to assure the efficacy of the treatment. Therapy monitoring with a systematic ASP activity measurement seems to be crucial to recognize silent inactivation and react to it properly.

Ammonia is a direct product of the reaction catalyzed by ASP. There are few studies concerning the ammonia concentration during a treatment with ASP [13–17]. A significant increase of ammonia levels after an intravenous ASP administration was described [13, 14, 16]. In some patients, symptomatic hyperammonemia occurs, especially after PEG-ASP, probably due to its prolonged half-life [13].

The ammonia measurement, as an easily available, fast, and cheap test, may be useful as a surrogate parameter of ASP activity. The aim of the study was to assess the clinical relevance of ammonia concentration during the ASP therapy.

## 2. Patients and Methods

Between June 2005 and October 2008, 97 children with newly diagnosed ALL started treatment with the international program ALLIC 2002 in the Department of Pediatric Oncology and Hematology of the Children's University Hospital in Krakow, Poland. Eighty-seven patients had blood samples available for the ASP activity and ammonia concentration monitoring.

There were 45 boys (52%) and 42 girls (48%), aged from 1.2 to 17 (median: 6, mean: 7; standard deviation/SD/: 4.3 years). 71 patients (81.6%) were diagnosed with common B-ALL (cALL), 3 (3.4%) with pro-B ALL, 2 (2.3%) with transitional ALL, and 11 (12.7%) with T-ALL. Among 71 patients with cALL, 25 were in the standard risk group (SRG), 31 in the intermediate risk group (IRG), and 15 in the high risk group (HRG). One child with proB-ALL and 2 with transitional ALL were in SRG, 2 patients with proB-ALL and 7 with T-ALL in IRG, and 4 with T-ALL in HRG.

All patients received native* E. coli* ASP (Asparaginase Medac, Medac Gesellschaft für klinische Spezialpräparate mbH, Hamburg, Germany) during induction (Protocol I), reinduction (Protocol II and III), and HR blocks (Table 1).

The blood samples for ASP activity and ammonia concentration were collected before each ASP administration during induction (3 days after the preceding administration). Serum for ASP activity evaluation was frozen at −80°C until the analysis was carried out. ASP activity was evaluated with MAAT test (Medac Asparaginase-Aktivitäts-Test, Medac Gesellschaft für klinische Spezialpräparate mbH, Hamburg, Germany). Blood for ammonia evaluation was taken before and 24 hours after ASP administrations to heparinized capillary tubes (lithium heparin) and transported on ice to the laboratory where immediately tested to eliminate the effect of ASP activity in vitro. In all patients, the activity of transaminases was monitored to recognize hepatotoxicity. All analyses were performed in the Department of Clinical Biochemistry in the Polish-American Institute of Pediatrics (Jagiellonian University Medical College, Krakow, Poland).

Statistical analyses were performed with the STATISTICA 8 software. Correlations were assessed with Spearman correlation analysis. Mann-Whitney* U* test was used to compare the differences between the two groups. The prognostic capacity of ammonia tests was evaluated in terms of the receiver-operating characteristic (ROC) curve. It was assumed that a test with an area under curve (AUC) of more than 0.9 has high accuracy, AUC from 0.7 to 0.9 indicates moderate accuracy, from 0.5 to 0.7, low accuracy, and below 0.5, a chance result [18].

## 3. Results

The activity of ASP was analyzed in 374 samples. The mean ASP activity was 257 (standard deviation (SD): 168) and median was 248 (range: 0–2063) IU/L. Activity below 100 IU/L was noted in 59 (16%) samples in 19 (21%) patients. In 15 (4%) samples, activity was undetectable (<30 IU/L). It was noted in 7 (8%) children.

The ammonia concentration was assessed in 535 samples taken before the administration of ASP (3 days after the preceding dose) and in 536 samples taken 24 hours after ASP. The mean ammonia concentration before ASP administration was 38 *μ*mol/L (SD: 28 *μ*mol/L) and median was 32 *μ*mol/L (range: 1.4–208 *μ*mol/L). One day after ASP administration, the mean ammonia concentration was 103 *μ*mol/L (SD: 64 *μ*mol/L) and median was 88 *μ*mol/L (range: 5–491 *μ*mol/L). The median increase of the ammonia concentration 24 hours after ASP administration with reference to the concentration before that ASP dose was 51 *μ*mol/L. We did not observe symptoms of hyperammonemia.

There was a positive correlation between the ammonia concentration 24 hours and 3 days after ASP administration and the activity of the drug 3 days after the administration (*R* Spearman: 0.34 and 0.44, resp.; *P* < 0.0001).

For each patient, the mean value of ammonia concentrations (separately for the measurements performed 24 hours and 3 days after the drug administration) and the mean value of ASP activities were calculated. A significant correlation between the mean values of the ammonia concentration and the mean value of ASP activity was found (*R* Spearman 0.41; *P* < 0.0001 and 0.56 *P* < 0.0001 for ammonia measurements performed 24 hours and 3 days after ASP administration, resp.). The mean ammonia concentration was lower in patients with decreased ASP activity (<100 IU/L) in at least one measurement than in children with the therapeutic activity of the enzyme in all tested samples (ammonia 24 hours after ASP: median 70 *μ*mol/L and 106 *μ*mol/L, resp.; ammonia 3 days after ASP: median 29 *μ*mol/L and 42 *μ*mol/L, resp.; *P* < 0.00001).

**Figure 1:**
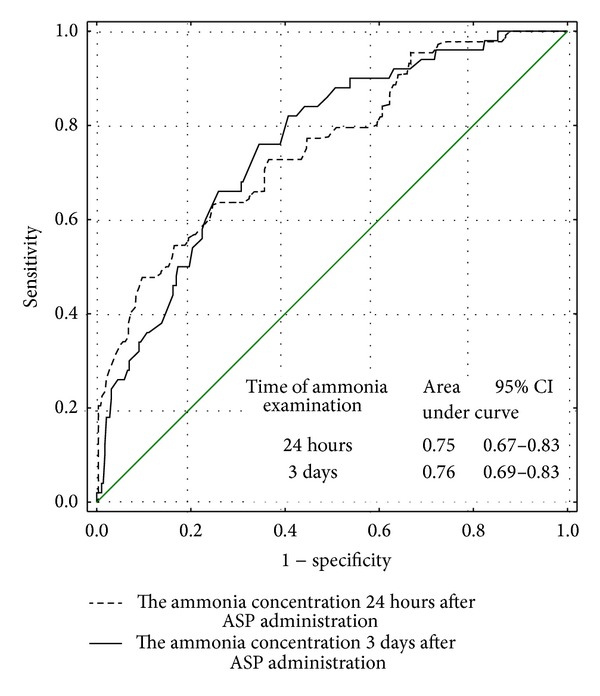
The usefulness of the ammonia concentration measured 24 hours and 3 days after ASP administration in detection of low (<100 IU/L) ASP activity—ROC curve.

**Figure 2:**
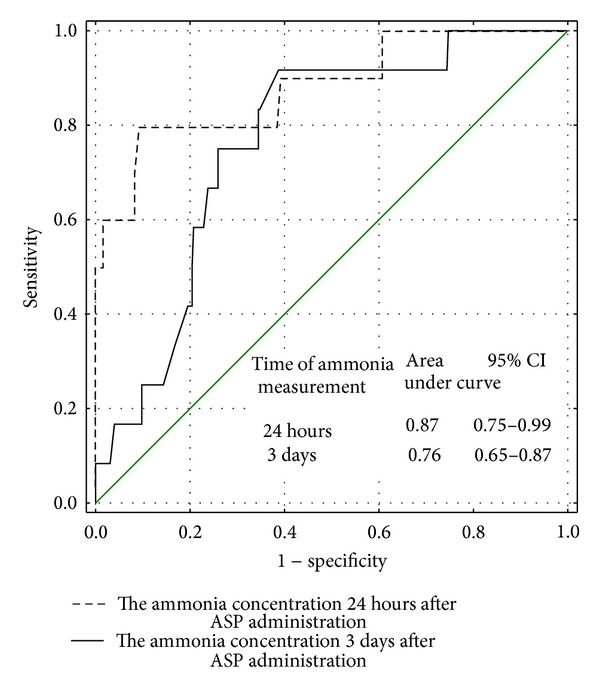
The usefulness of the ammonia concentration measured 24 hours and 3 days after ASP administration in detection of undetectable (<30 IU/L) ASP activity—ROC curve.

On the basis of a ROC curves analysis, the usefulness of ammonia concentration in detection of low and undetectable ASP activity was stated (AUC for ammonia concentration 24 hours and 3 days after ASP administration: 0.75 and 0.76, resp.; for low ASP activity; 0.87 and 0.76, resp.; for undetectable ASP activity—Figures 1 and 2).

The optimal cut-off point for detection of low ASP activity was 64 *μ*mol/L and 29 *μ*mol/L for the ammonia concentration 24 hours and 3 days after ASP administration, respectively. For undetectable ASP activity, the ammonia cutoff was 38 *μ*mol/L (24 hours after ASP) and 24 *μ*mol/L (3 days after ASP). The parameters assessing the value of the tests recognizing low and undetectable ASP activity for different cut-off points of ammonia concentration are shown in Tables 2 and 3.

**Figure 3:**
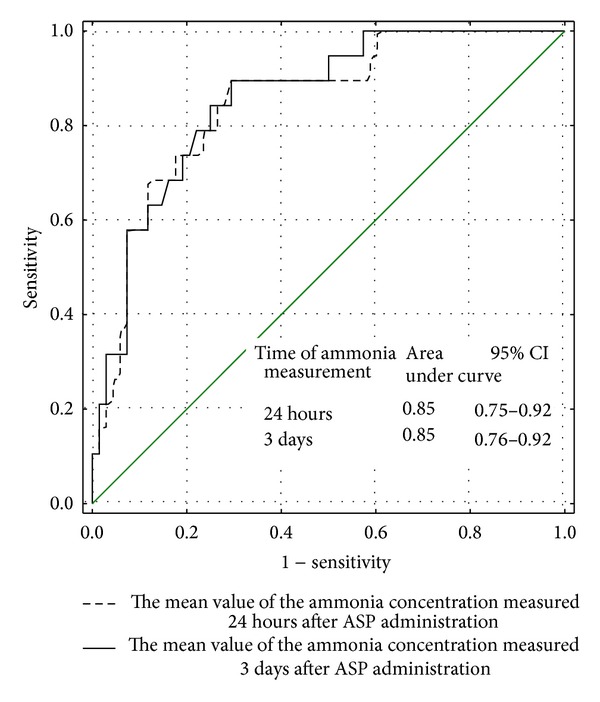
The usefulness of the mean value of the ammonia concentration measured 24 hours and 3 days after ASP administration in identifying the patients with low (<100 IU/L) ASP activity in at least one examination—ROC curve.

**Figure 4:**
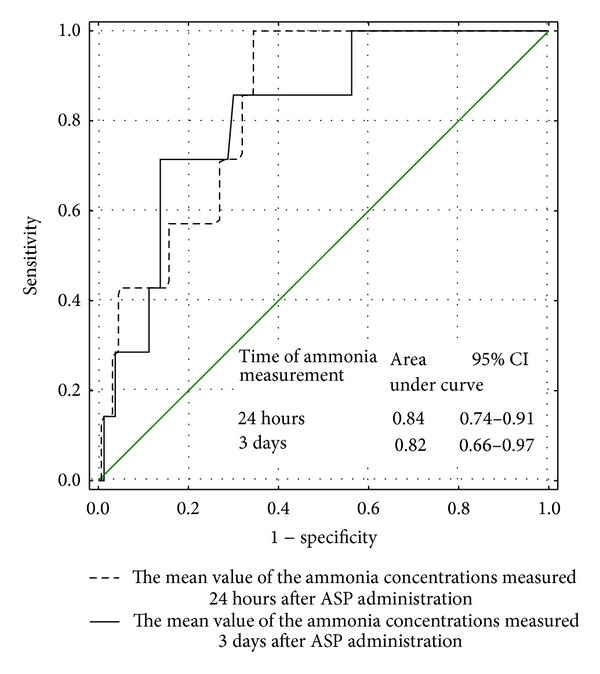
The usefulness of the mean value of the ammonia concentration measured 24 hours and 3 days after ASP administration in identifying the patients with undetectable (<30 IU/L) ASP activity in at least one examination—ROC curve.

The mean value of ammonia concentration during induction was found to be useful to distinguish between the patients with the therapeutic ASP activity in all measurements during induction and the patients with low or undetectable ASP activity in at least one determination during induction (AUC for low and undetectable ASP activity: 0.85 and 0.84, resp.) (Figures 3 and 4).

To identify the patients with low and undetectable ASP activity, the optimal cutoff points for the mean ammonia concentration determined 24 hours after ASP administration were 91 *μ*mol/L and 90.5 *μ*mol/L, respectively, and for the mean ammonia concentration determined 3 days after ASP administration, 36 *μ*mol/L and 29 *μ*mol/L, respectively. The parameters assessing the ability of the tests to identify the patients with low and undetectable ASP activity for different cut-off points of the mean ammonia concentration are shown in Tables 4 and 5.

Hepatotoxicity grade III and IV WHO was found in 5 patients. The ammonia concentration in this group of patients did not differ significantly from the ammonia concentration in the patients without severe hepatotoxicity. No correlation between the ammonia concentration and transaminases activity was found.

## 4. Discussion

ASP is among the most important agents in the therapy of ALL. Treatment schedules for each preparation assure clinical efficacy in the majority of patients. Taking into account the risk of ASP inactivation by antibodies, the therapy monitoring with ASP activity measurements is very important [5, 6, 8].

Ammonia is a direct product of the reaction catalyzed by ASP and its plasma concentration can be easily measured as a routine, inexpensive laboratory test. Hyperammonemia is a well-known complication of ASP administration [13–15, 19]. Steiner et al. described ammonia fluctuations in 10 patients treated with ASP, up to sevenfold above the normal values 1 day after ASP administration and slow returning to the normal level within 2 consecutive days, but the authors did not monitor ASP activity [16]. Watanabe et al. found that ex vivo ammonia production well correlated with ASP activity, but only 5 patients were enrolled to the study and 23 samples were tested [17]. To the authors' knowledge, till now, there has been no publication describing a correlation between plasma ammonia level and ASP activity in larger cohort.

Ammonia produced by ASP reaction is removed in the liver by incorporation into the urea cycle, so the ammonia level may be influenced by a hepatic dysfunction. Therefore, we monitored the liver function in the studied patients. There were 5 patients with WHO grade III and IV hepatotoxicity, but their ammonia concentrations were not significantly higher than those of other patients. We did not find any correlation between the ammonia concentration and transaminase activity in our patients.

We analyzed the ammonia concentration 24 hours and 3 days after ASP administration during induction. The ammonia concentration correlated with ASP activity. The utility of ammonia measurements in recognizing low and undetectable ASP activity was assessed. On the basis of an ROC curves analysis, we found the moderate accuracy of the test. The optimal ammonia concentration thresholds to identify low and undetectable ASP activity were determined. The sensitivity and specificity of these tests were acceptable especially for undetectable activity of the enzyme, but the positive predictive values (PPV) were very low, which means a high percentage of false positive results.

A better test accuracy was found for the mean value of ammonia concentrations. An analysis of the ROC curve revealed that the mean value of ammonia concentrations can be useful to identify patients with low and undetectable ASP activity in at least one determination during induction. Using ammonia measurements, 89% of patients with low ASP activity and 70% of patients with the therapeutic ASP activity could be correctly recognized and among the patients with positive results (the mean ammonia level below the threshold), 46% would be those with low ASP activity. Taking into account the fact that low ASP activity occurred in 21% of the examined patients, false positive results would amount to 23% of all results. That would result in recognizing silent inactivation in the patient with the therapeutic enzyme activity and unwarranted switching to another ASP preparation. As all ASP preparations have similar efficacy and safety profile while used at proper doses [5, 8, 11], it would have mainly financial consequences, because of higher costs of the pegylated preparations or those derived from* Erwinia*.

A reduction of false positive results could be achieved by decreasing the threshold of ammonia level. An increase of false negative results is synonymous with more patients with unrecognized silent inactivation, at risk of an ineffective treatment and a symptomatic allergic reaction.

## 5. Conclusions

The ammonia concentration correlates with ASP activity. The lower the ammonia concentration is, the higher the probability of low ASP activity is. Although the ammonia concentration measurement is not accurate enough to replace a direct ASP activity assay, it can be helpful when a direct assay is not available. Identification of the patients with low and especially undetectable ASP activity “silent inactivation” with such an easily available measurement could help in making decisions about switching to another ASP preparation to ensure the efficacy of the treatment.

## Figures and Tables

**Table 1:** The protocols of the ALL IC 2002 program with L-ASPA administration.

Protocol I	5000 IU/m^2^/day—days: 12, 15, 18, 21, 24, 27, 30, and 33
Protocol II	10000 IU/m^2^/day—days: 8, 11, 15, and 18
Protocol III	10000 IU/m^2^/day—days: 1, 4, 8, and 11
HR blocks 1–3	25000 IU/m^2^/day—days: 6 and 11

**Table 2:** The parameters assessing the ability of the ammonia concentration to recognize low (<100 IU/L) ASP activity (95% CI in brackets).

	The ammonia concentration after ASP administration	
	24 hours ≤ 64 *μ*mol/L	3 days ≤ 29 *μ*mol/L
Sensitivity	0.64 (0.51–0.76)	0.76 (0.62–0.87)
Specificity	0.75 (0.72–0.76)	0.65 (0.59–0.71)
Positive predictive value	0.30 (0.24–0.35)	0.27 (0.22–0.30)
Negative predictive value	0.93 (0.90–0.95)	0.94 (0.91–0.96)

**Table 3:** The parameters assessing the ability of ammonia concentration to recognize undetectable (<30 IU/L) ASP activity (95% CI in brackets).

	The ammonia concentration after ASP administration	
	24 hours ≤ 38 *μ*mol/L	3 days ≤ 24 *μ*mol/L
Sensitivity	0.80 (0.50–0.94)	0.75 (0.47–0.91)
Specificity	0.90 (0.89-0.90)	0.74 (0.73–0.75)
Positive predictive value	0.21 (0.13–0.25)	0.09 (0.06–0.12)
Negative predictive value	0.99 (0.98–1.0)	0.99 (0.97–1.0)

**Table 4:** The parameters assessing the ability of the mean value of the ammonia concentrations to recognize patients with low ASP activity in at least one examination (2 cut-off points, 95% CI in brackets).

	The mean value of the ammonia concentration			
	24 hours after ASP administration		3 days after ASP administration	
	≤46 *μ*mol/L	≤91 *μ*mol/L	≤20 *μ*mol/L	≤36 *μ*mol/L
Sensitivity	0.17 (0.04–0.41)	0.89 (0.67–0.99)	0.22 (0.06–0.48)	0.89 (0.67–0.99)
Specificity	0.99 (0.92–1.0)	0.70 (0.58–0.81)	0.99 (0.92–1.0)	0.70 (0.58–0.81)
Positive predictive value	0.75 (0.19–0.99)	0.46 (0.28–0.63)	0.80 (0.28–0.99)	0.46 (0.30–0.60)
Negative predictive value	0.82 (0.72–0.89)	0.96 (0.86–0.99)	0.83 (0.73–0.90)	0.96 (0.89–0.99)

**Table 5:** The parameters assessing the ability of the mean value of the ammonia concentrations to recognize patients with undetectable ASP activity in at least one examination (2 cut-off points, 95% CI in brackets).

	The mean value of the ammonia concentration			
	24 hours after ASP administration		24 hours after ASP administration	
	≤53 *μ*mol/L	≤90.5 *μ*mol/L	≤14 *μ*mol/L	≤29 *μ*mol/L
Sensitivity	0.43 (0.10–0.82)	1.0 (0.59–1.0)	0.14 (0.01–0.58)	0.71 (0.29–0.96)
Specificity	0.96 (0.89–0.99)	0.66 (0.55–0.76)	0.99 (0.93–1.0)	0.86 (0.77–0.93)
Positive predictive value	0.50 (0.12–0.88)	0.21 (0.08–0.38)	0.50 (0.13–0.99)	0.31 (0.11–0.59)
Negative predictive value	0.95 (0.88–0.99)	1.0 (0.93–1.0)	0.93 (0.85–0.97)	0.97 (0.90–1.0)
